# On the Features of Composite Coating, Based on Nickel Alloy and Aluminum–Iron Bronze, Processed by Direct Metal Deposition

**DOI:** 10.3390/ma14040957

**Published:** 2021-02-18

**Authors:** Eugene E. Feldshtein, Oleg Devojno, Marharyta Kardapolava, Nikolaj Lutsko, Justyna Patalas-Maliszewska

**Affiliations:** 1 Institute of Mechanical Engineering, University of Zielona Góra, Prof. Z. Szafrana 4, 65-516 Zielona Góra, Poland; E.Feldsztein@ibem.uz.zgora.pl; 2 Faculty of Mechanical Engineering, Belarusian National Technical University, Khmelnitsky Street 9, Building 6, 220013 Minsk, Belarus; devoino-o@mail.ru (O.D.); margokardo@mail.ru (M.K.); nilucko@tut.by (N.L.)

**Keywords:** additive manufacturing, composite coating, Ni-based alloy, Fe–Al bronze, microstructure, mechanical properties, tribological behavior

## Abstract

In recent years, additive manufacturing technologies have become increasingly widespread with the most intensive development being direct metal deposition (DMD), alloys, and ceramic materials on a metal substrate. This study shows the possibilities of the effective formation of coatings, based on heterogeneous metal alloys (Ni-based alloy and Fe-Al bronze) deposited onto 1045 structural steel. Changes in the microhardness, the microstructure, and the tribological properties of the composite coating, depending on the laser spot speed and pitch during DMD processing, have been considered. It was revealed that if the components of the composite coating are chosen correctly, there are possible DMD conditions ensuring reliable and durable connection between them and with the substrate.

## 1. Introduction

In the past few years, the use of additive manufacturing (AM) technologies has been growing intensively to form elements using different metals, alloys, and ceramics. Research into AM processes considers both the general problems of the formation of products as well as the formation of microstructures, the mechanical and strength characteristics of the material formed, its wear resistance, and its resistance to corrosion, etc.

Choi and Chang [1] studied the direct metal deposition (DMD) process of H13 steel and the characteristics of the products. It was shown that the range of processing parameters is rather narrow for the production of samples with dimensional stability and microstructure integrity. El Cheikh et al. [2] investigated the process of the direct laser fabrication (DLF) of products made of 316 L steel, studying, for example, the effect on the laser spot speed and the different laser shift-distances between two successive layers. Interactions between the process parameters were found. Ma et al. [3] studied the shapes and characteristics of parts consisting of 316 L steel using DLF technology. It was shown that a precision sample with a homogeneous surface can be obtained with a small laser spot diameter and higher laser spot speed. Gharbi et al. [4] studied the DMD process of a Ti-based alloy and revealed the outcome of laser beam power, laser spot speed, and powder flow on changes of the geometry and structure of the element. Yu et al. [5] completed an analysis of the resistance to cracking and the microstructure and mechanical properties of elements produced with stainless steels by the laser metal deposition (LMD) method. Yu et al. [6] investigated elements of different wall thicknesses produced by LMD similar to the 718 alloy and compared the structure of the material with that produced by traditional methods. Caiazzo et al. [7] studied the DMD process using a Ni-based superalloy powder and showed that, when compared with traditional methods, minimal billet distortion, a reduction of the heat affected zone, and a better quality of surface were observed. Special attention was given to powder feeding methods and the peculiarities of single track formation. Govekar et al. [8] described a DMD head that used the axial feeding of pure Ni and varied the intensity of the distribution of the laser beam on the surface of samples made of 304 steel. The stability of the deposition process and the strength of the material deposited were achieved. Akbari et al. [9] described the production of thin-walled and block samples from stainless steel, with short and long time depositing intervals. The deposition conditions affected the form and size of the grains and the ultimate tensile strength and hardness of the material.

Ju et al. [10] considered the process of the laser cladding of high chromium content Fe-based powders on 42CrMo steel and showed that the main factors influencing the width and depth of the deposited layer were the power and speed of the laser. Mazzucato et al. [11] described the DMD process of 316 L powder steel and investigated the influence of deposition parameters on sample geometry. Haley et al. [12] studied the laser-directed energy deposition (LDED) method in which a powder of 316 L stainless steel was injected into the melted metal pond. It was revealed that due to surface tension, the powder particles are trapped on the surface and float before melting, which increases surface roughness. Froend et al. [13] described solidification conditions upon the laser deposition of an Al–Mg alloy wire and showed that differences in microstructure could be achieved by adjusting the radiation of the laser beam. Zhao et al. [14] used the DLD method to produce samples from 24CrNiMo steel and found that changes in microstructure, texture, and mechanical properties can be affected by changes in interlayer intervals of time and scanning strategy. Hu et al. [15] studied the effect of temperature on the static recrystallization and plasticity of the Inconel 625 super-alloy, produced by the DED. Kies et al. [16] considered the formation peculiarities, the mechanical properties, and the possibilities of preventing defects in the DED of high-manganese steels. Cui et al. [17] studied the LMD process of 12CrNi2 steel and found relationships “process—microstructure—properties”. Kang et al. [18] analyzed 24CrNiMo steel samples produced by the LMD method, particularly the micro-structure, grain size, and intensity of texture of single and multi-layers. Yu et al. [19] studied the DMD process of 17-4 PH steel and described the formation of porosity, microstructure, and roughness of the upper surface of the deposited layer. Rankouhi et al. [20] compared the Selective Laser Melting (SLM) and Direct Energy Deposition (DED) processes of 316 L steel products and showed that SLM provided higher yield strength than DED due to more homogeneous grain sizes and denser dislocation structures. Saboori et al. [21] evaluated the effect of the conditions of the deposition process on the microstructure and the mechanical characteristics as well as the residual stresses of 316 L samples in the DED process and showed that the cooling rate first decreased and then increased again at the last layers due to their increased distance from the substrate. Mendagaliyev et al. [22] studied the DLD process of elements made of 09CrNi2MoCu steel powder and found regularities in the influence of the technological parameters on the material formed. Wang et al. [23] used the LMD method in the manufacture of samples made of a K648 superalloy and studied the microstructure and mechanical properties of the layer deposited. It was found that microstructures in the lower and middle areas of the layer differ significantly from those in the upper area and that the tensile strength limit is anisotropic. Bodner et al. [24] synthesized a multi-layered structure based on alternate layers of IN 625 alloy and 316 L stainless steel on the 316 L steel substrate and showed that the periodic appearance of IN 625 and 316 L zones correlated with increasing and decreasing hardness and decreasing and increasing compressive stresses. Lian et al. [25] researched the effect of the different parameters of the multi-track laser cladding of curved surfaces made with W6Mo5Cr4V2 steel powder deposited on 1045 steel. The increase in laser power and gas flow, with a simultaneous decrease in overlapping ratio, reduced the incomplete layer fusion and provided smaller pore sizes in the cladding layer. Oh et al. [26] examined the conditions for the formation of cracks at the layer interface at 316 L steel after DMD. The formation of macrocracks was fixed upon deposition with the minimum of excess, whereas only microcracks were observed when it increased. Pant et al. [27] defined and discussed the geometrical characteristics and mechanical properties of single-layer and multi-layer walls when DMD processing with a coaxial-powder flow of 316 L steel, depending on the laser power, the laser spot speed, the intensity of the powder feeding, and overlapping factors. Ni et al. [28] researched the influence of coaxial nozzle parameters on gas flow and the powder distribution on the surface formed by the DMD method and showed that the correct choice of these parameters ensures good morphology and quality of the deposited layer.

Analyzing the studies described above, it should be emphasized that the powder and substrate materials were mostly either authentic or sufficiently close, for example, nickel superalloy and stainless steel. However, there is practically no information about the peculiarities of joining heterogeneous materials of the coating layer and substrate. The main objective of the present research is to analyze the possibility of joining a two-component coating layer of high quality with middle steel substrate.

## 2. Materials and Methods

### 2.1. Laser Machine Used

The self-made laser machine was used based on CO_2_ “Komet 2” laser of 1 kW power. The lens that provided the coaxial powder feed was applied, with three powder flows being fed into the cladding zone. Detailed description of the lens design is described in [29].

### 2.2. Materials

The 1045 steel, which is one of the most common structural steels, was used as a substrate. After shot blasting, a two-component coating layer was clad onto the substrate’s surface. To form this layer, powders of the 12N-01 nickel-based alloy and aluminum–iron bronze (Table 1) were used.

The hardness of the Ni-based alloy was equal to HRC 35–40 and the hardness of the bronze used was equal to HB 65–70. It should be said that the hardness of the Ni-based alloy was significantly lower compared to the 316 L steel powders [30]. It was found earlier [31,32] that powders with a granulation of 20–80 µm were optimal for deposition. The compressed air was used as a transporting gas during processing.

### 2.3. Preparation of Samples

**Figure 1:**
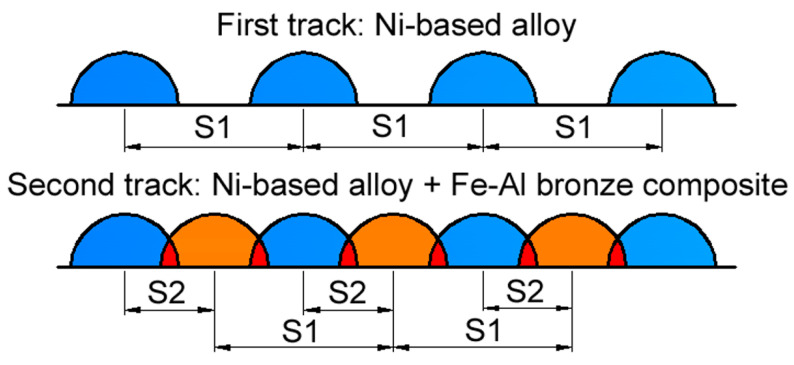
The composite coating deposition scheme.

The Ni–Al–Fe composite layer was formed as follows: first, tracks of nickel alloy were applied to the steel substrate with pitches S_Ni_ = 1.8, 2.4, and 3 mm while in the second step, Al–Fe bronze tracks were deposited between them. The pitch between nickel-based alloy and bronze tracks was S_Br_ = 0.9, 1.2, and 1.5 mm, respectively (here and hereafter Ni means the Ni-based alloy and Br means Fe–Al bronze). The difference in pitch size provided optimal overlapping, in which the bronze track was deposited exactly between the hollows of the two adjacent Ni alloy tracks. Coatings were formed at combinations of laser spot speeds V_Ni_/V_Br_ = 80/150 mm/min; V_Ni_/V_Br_ = 120/200 mm/min and V_Ni_/V_Br_ = 160/250 mm/min. The focal distance was 200 mm, the diameter of the laser spot was 1.0 mm, and the distance between the substrate surface and the nozzle was 10 mm. The nominal power density of process studied was equal to ~130 kW/cm^2^. The coating deposition scheme is shown in Figure 1.

Samples for the tribological tests were prepared under laser spot speeds: V_Ni_/V_Br_ = 100/150 mm/min, 110/160 mm/min, 120/170 mm/min, and 130/180 mm/min. The ratio of pitches between the Ni-based alloy and bronze tracks was taken as constant, S_1_/S_2_ = 2.4/1.2 mm. Samples for testing under conditions of dry friction were blocks with sizes 20 × 30 × 8 mm^3^. Samples for boundary lubrication tests were discs 50 mm in diameter and 12 mm thick. After DMD processing, the coating surfaces were ground to provide a roughness parameter of Ra 1.6 µm.

### 2.4. Measuring Equipment

A Micromet II tester (Buehler, Esslingen, Germany) was used to control the microhardness of the coating layer and a 100 g load was used. Analysis of the microstructures and scanning electron microscopy (SEM) was completed using a “Tescan Mira” scanning electron microscope (Tescan, Brno, Czech Republic). Tribological testing was fulfilled under dry friction conditions using a self-made tester according to the “ball–plate” scheme. The results were automatically recorded in the co-ordinates “coefficient of friction—path of friction”. The counter-body was a ball, 3 mm in diameter, and made of hardened bearing steel; the speed of the reciprocating motion was 5 mm/s; the length of a single pass was equal to 15 mm; the total friction path was 10 m; and the normal load was equal to 0.2 N. Wear tests under boundary friction conditions were carried out using a self-made tester acting according to the scheme “bushing end—plate”. The counter-body was a cemented carbide bushing, 16 mm in diameter with a wall thickness of 1.5 mm, which rotated with a line speed of ~1.8 m/s. The specimen was a coated stationary disk. Machine oil was dripped to the test area. An estimation of the volume of wear was made on the basis of measurements of the width and depth of the wear groove using a “Proton 130” roughness tester (JSC Plant Proton, Moscow, Russia). A graphical analysis of the results obtained was carried out using Statistica v13.3 software (StatSoft Polska Sp. z o.o., Kraków, Poland).

## 3. Results and Discussion

### 3.1. The Microstructure of Composite Coating

**Figure 2:**
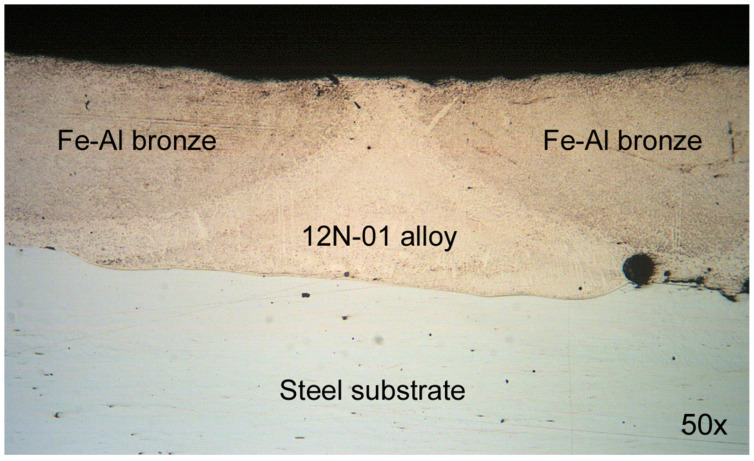
The fragment of coating deposited under conditions: laser spot speeds *V*_Ni_/*V*_Br_ = 160/250 mm/min and laser spot pitches *S*_1_/*S*_2_ = 2.4/1.2 mm.

Figure 2 shows a fragment of a composite coating layer consisting of two bronze tracks with a Ni alloy track between them deposited on a steel substrate.

The micro-structure features of the composite coating layer were studied in the following areas:

- Area I—the central transition zone between the Ni alloy track and the two bronze tracks;
- Area II—transition zone between the bronze and Ni alloy tracks; and
- Area III—transition zone between the Ni alloy track and the substrate.

**Figure 3:**
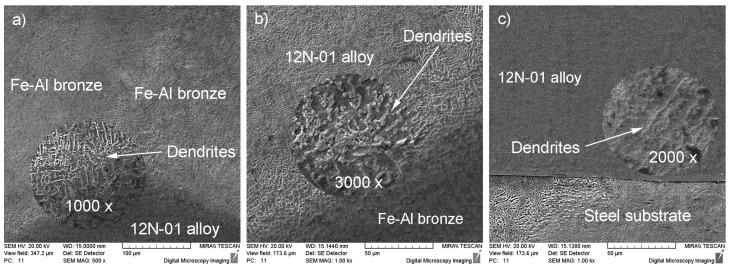
Microstructures of the areas analyzed: (**a**) Area I, (**b**) Area II and (**c**) Area III.

SEM analysis of the microstructures in Areas I—III gives credence to the claim that the microstructure of the whole composite layer, deposited on the steel substrate, was very uniform. In particular, the bronze tracks deposited on the Ni alloy track had a dendritic structure with axes at an angle of 45° to the surface, indicating a high crystallization speed. The Ni alloy structure is globular and the eutectic component of the alloy is clearly distinguishable therein (Figure 3).

There was no mutual diffusion zone at the interface between the composite deposited layer and the steel substrate due to the absence of significant sample heating and the thorough penetration of the deposited layer. The thickness of the transition zone decreased as the deposition rate of the nickel alloy on the surface of the steel substrate increased. In this case, continuous solid solutions of iron in nickel had no time to form, but the chemical bonds were significant enough and the sizes of the structural components decreased. The dendrites had only first-order axes and passed into the quasi-eutectic state. In DMD processing, the chromium in the boundary layer is distributed discretely, which indicates the formation of Cr carbides during the deposition process, as described in [30]. Diffusion of Al and Cu into the Ni-based alloy was not observed. Iron, present in both the Ni alloy and the bronze, was distributed uniformly throughout the thickness of the coating layer, although in lower amounts compared to its initial concentration. Nickel, which was the base of the alloy under study, penetrated into the bronze in a sufficiently large amount in all cases.

### 3.2. The Effect of Direct Metal Deposition (DMD) Parameters on the Micro-Hardness of the Composite Coating

**Figure 4:**
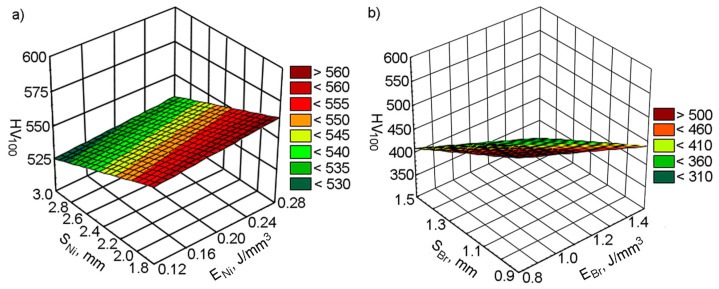
The effect of the energy density and laser spot pitch on the microhardness of the deposited tracks: (**a**) nickel-based alloy; (**b**) bronze.

The relationships between microhardness inside deposited tracks and DMD conditions were observed. It was revealed that the average microhardness of the Ni alloy decreased when the laser spot speed increased, independently of the pitch value. This is due to changes of the energy density that enters the coating when the laser spot moves along the simple track direction (Figure 4).

At nickel alloy and bronze cladding speeds of *V*_Ni_/*V*_Br_ = 80/150 mm/min, the energy density in the deposited layer was enough to form a comparatively large molten pond. Herewith, the solidification rate was sufficiently high to shape a structure with high microhardness. When cladding speeds increased to *V*_Ni_/*V*_Br_ = 120/200 and *V*_Ni_/*V*_Br_ = 160/250 mm/min, the energy density in the coating layer dwindled. Under such conditions, the melt pond volume decreases and the solidification speed increases if the same amount of powder is fed into the laser pond. As a consequence, the incomplete fusion of materials on the tracks’ borders starts to play an increasing role and results in decreasing the average microhardness of the Ni alloy in the coating layer.

The influence of the pitch on the average microhardness of nickel alloy is more complicated. The highest micro-hardness at all laser spot speeds was overseen with pitches *S*_1_/*S*_2_ = 2.4/1.2 mm. Due to the good heating and cooling conditions of the adjacent tracks, terms for the formation of an optimum structure were created, which allowed for the microhardness to increase. Reducing the track pitch to *S*_1_/*S*_2_ = 1.8/0.9 mm resulted in the material overheating due to the repeated heating of neighboring tracks; this increased grain size, which caused a decrease in the microhardness. When the pitch grew to *S*_1_/*S*_2_ = 3.0/1.5 mm, reheating during the cladding of neighboring tracks had an insignificant outcome. However, the incomplete fusion of material, appearing on the grain boundaries, provided a decrease in the micro-hardness of the nickel alloy. The average microhardness of the bronze in the coating layer does not depend, ultimately, on the cladding speed. The effect on the track pitch was similar to that of the 12N-01 alloy. A higher microhardness was registered with a cladding pitch of *S*_1_/*S*_2_ = 2.4/1.2 mm. When decreasing the pitch value to *S*_1_/*S*_2_ = 1.8/0.9 mm and increasing it to *S*_1_/*S*_2_ = 3.0/1.5 mm, the microhardness of the bronze decreased. This can be explained by the change in the heating and cooling conditions.

It should also be marked that no interactions between pitch and energy density values were registered when DMD processing.

### 3.3. The Microhardness Characteristics of the Coating Layer

**Figure 5:**
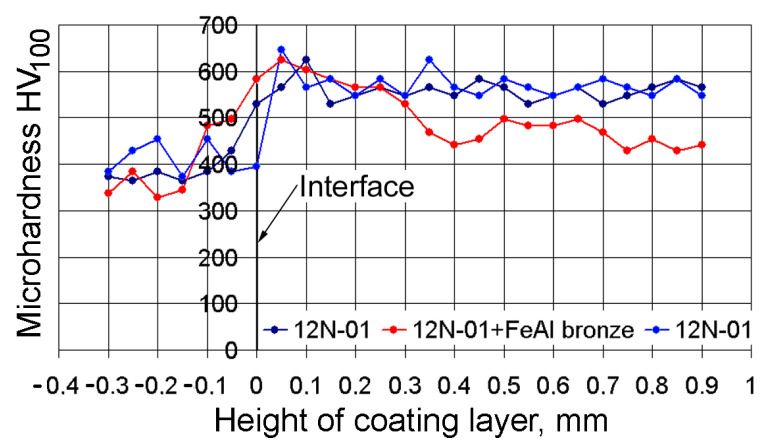
The distribution of micro-hardness by the depth of the coating layer, deposited under conditions V_Ni_/V_Br_ = 120/200 mm/min and S_1_/S_2_ = 2.4/1.2 mm.

The character of the distribution of microhardness inside the coating layer is shown in Figure 5. There was a considerable growth in microhardness on the interface of the substrate and on the deposited layer of coating. The transition zone into the substrate body was not much thicker than 0.3 mm. However, at a distance of 0.1 mm from the substrate surface, the microhardness of the deposited layer of nickel alloy stabilized throughout the entire height of the coating. This indicates a good mixing of the materials in the molten metal pond and the uniform heating of the single track of the material. Microhardness of the nickel alloy tracks was on average HV_100_ = 550.

The distribution of micro-hardness in the bronze track was slightly different. Microhardness growth was observed in the surface layer of a coating as well as in the tracks formed of nickel alloy. Such growth proceeds up to a distance of 0.1 mm from the interface with the steel substrate; the average microhardness in a track was HV_100_ = 550 and was equal to the microhardness of the nickel alloy. At a distance of 0.3 mm from the interface the microhardness decreased to HV_100_ = 450 and remained at this level at all coating thicknesses. It is fashionable to assume that when the bronze track is deposited, it is uniformly heated and well mixed with the nickel alloy in the bath of molten metal. The average microhardness inside the bronze track was HV_100_ = 450.

**Figure 6:**
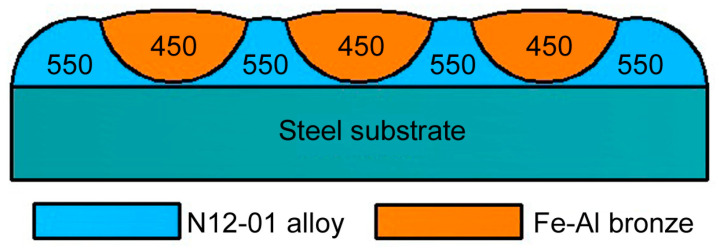
Changes in microhardness in the coating in the direction parallel to the surface of the substrate.

The general character of the change in the microhardness of the coating layer in the direction parallel to the substrate’s surface is shown in Figure 6. Such layers represent a kind of “grid” of the 12N-01 alloy with an average microhardness of HV_100_ = 550 with bronze having an average microhardness of HV_100_ = 450 (Figure 6).

**Figure 7:**
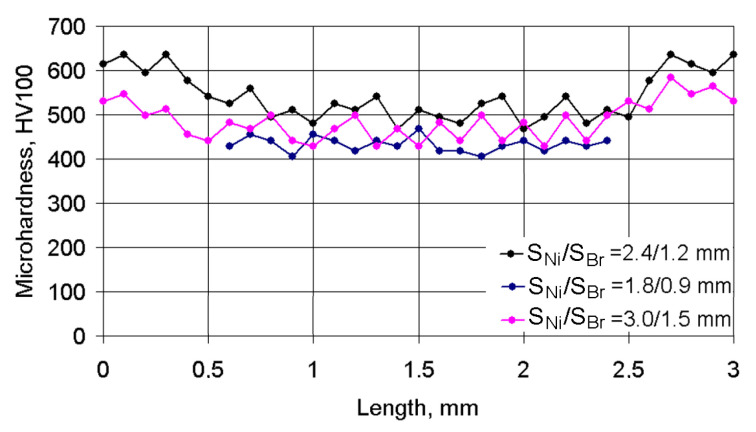
Changes in microhardness in the cross section of three neighboring deposited tracks under a laser spot speed of V_Ni_/V_Br_ = 120/200 mm/min and different pitches.

The character of the changes in microhardness in the cross section of three neighboring deposited tracks is shown in Figure 7. The starting point for measuring corresponded to the middle of the left track of the Ni alloy, while the final measurement point corresponded to the middle of the right track of the Ni alloy. The 1.5 mm co-ordinate corresponded to the middle of the central bronze track. It can be clearly seen in the figure that some periodicity changes in microhardness were observed for depositing pitches *S*_1_/*S*_2_ = 2.4/1.2 mm and *S*_1_/*S*_2_ = 3.0/1.5 mm. First, when measurements were made in a 12N-01 alloy track, the average microhardness of the coating was at a level of HV_100_ = 630–530. When measurements were carried out in the Fe–Al bronze track, microhardness was reduced to HV_100_ = 480–350. When measurements were made again in the 12N-01 alloy track, average microhardness increased again up to HV_100_ = 630–530. For smaller pitches of deposited tracks equal to *S*_1_/*S*_2_ = 1.8/0.9 mm, the microhardness was stable, which was connected with a more uniform mixing of the molten metals in the laser pond.

### 3.4. Tribological Behavior

It was noted above that tribological tests were carried out under both dry and boundary friction conditions. This approach allowed the efficiency of using composite material to be evaluated, based on the deposition of nickel alloy and bronze by the DMD method, in order to regenerate worn parts.

**Figure 8:**
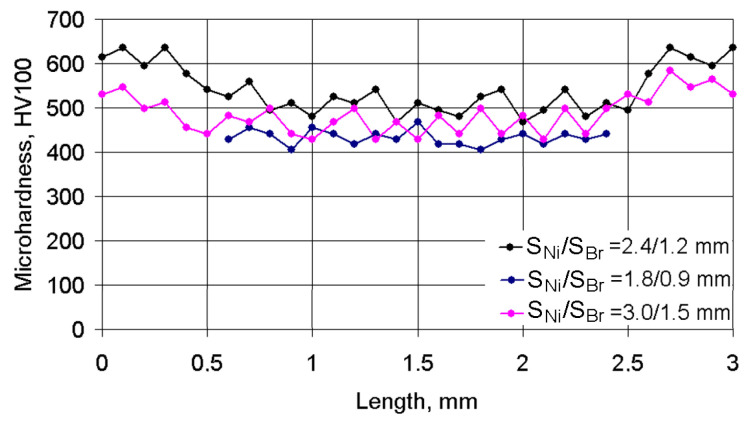
Dependences of the coefficient of friction (CoF) of the composite coating on the friction path under dry friction conditions.

Dependences of the coefficient of friction (CoF) on the friction path for composite coatings are shown in Figure 8. The running-in time (about 2 min) was characterized by low values of the CoF under dry friction conditions, less than 0.20. A gradual increase in the CoF up to 0.4–0.45 was registered for the stable wear period, after which there was a period of accelerated wear, in which CoF increased (deposition speed of 100/150 mm/min), or decreased (deposition speed of 130/180 mm/min)

**Figure 9:**
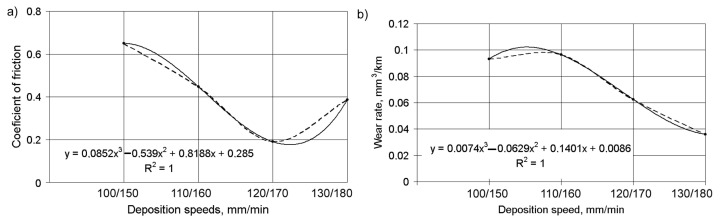
The dependence of the CoF (**a**) and the wear rate (**b**) of the composite coating under dry friction conditions on the deposition speed (dotted line—empirical data, solid line—approximation equation).

The changes in the CoF were caused by a number of factors; these include the energy density in the laser pond, features of subsequent cooling and hardening of the two-component layer, formation of its microstructure, and changes in microhardness. At a cladding speed of 100/150 mm/min, the energy density going to the DMD layer was high enough and a coarse-grained microstructure with reduced microhardness was formed in it. Such a structure promotes the transfer of bronze grains to the surface of the counter-body and the CoF increases. As the deposition speed increases, the energy density decreases and the cooling rate of the coating material increases. As a result, an increasingly fine-grained microstructure with increased microhardness is formed and the friction coefficient decreases. However, with any further increase of the deposition speed up to 130/180 mm/min, the CoF increases again. Under such conditions, the energy density in the cooled coating becomes insufficient, despite a further increase in the cooling speed of the molten material and the formation of an increasingly fine grain structure. This leads to poor adhesion of the track material at the grain boundaries, reduces microhardness, and promotes the chipping of particles from both boundary layer components. The CoF increases in this case. The general effect of the deposition speed on the CoF is shown in Figure 9a, and on the wear rate is shown in Figure 9b.

**Figure 10:**
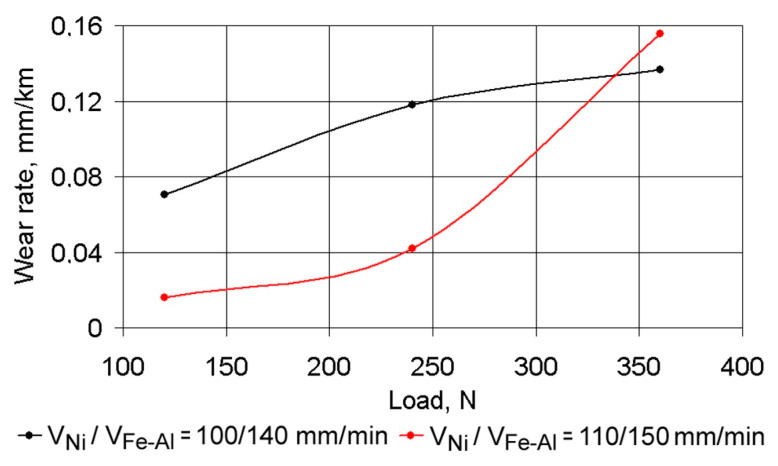
The dependence of the linear wear rate of the composite coating on a load under boundary lubrication conditions.

It is easy to see an inverse correlation between CoF values and wear rate under dry friction conditions. The best results can be achieved with Ni alloy DMD speeds of 120–125 mm/min and Fe–Al bronze DMD speeds of 170–175 mm/min. Dependences of the linear wear rate of the coating layer on the load under boundary friction conditions are shown in Figure 10. Under an increase in load from 120 up to 240 N, the wear rate increased more than in 2.2 times, irrespective of deposition speed. Thus, the wear rate with a laser spot speed of *V*_Ni_/*V*_Br_ = 100/140 mm/min is higher compared to a speed of *V*_Ni_/*V*_Br_ = 110/150 mm/min. In this case, an absence of adhesion was observed and the size of the load had almost no effect on the wear. However, increasing the load further (up to 360 N) with a layer depositing speed of *V*_Ni_/*V*_Br_ = 110/150 mm/min, increased the wear rate almost four times, thus indicating the appearance of adhesion. At the same time, for coatings deposited at a speed of *V*_Ni_/*V*_Br_ = 100/140 mm/min, the wear rate increased by 10–15% only, when the load increased up to 360 N. Such correlations can be explained by the peculiarities of the composite coating of the microstructure. Coatings deposited at a speed of *V*_Ni_/*V*_Br_ = 110/150 mm/min had a finer-grained and more wear-resistant structure.

This is significant at low and medium loads only. Under higher loads, the adhesion force on the grain boundaries in the single tracks of the coating material begins to play a major role. This force was higher for coatings deposited with *V*_Ni_/*V*_Br_ = 100/140 mm/min. In the layer deposited with *V*_Ni_/*V*_Br_ = 110/150 mm/min, under significant loads, the single track material begins to break down, leading to adhesion and accelerated wear.

**Figure 11:**
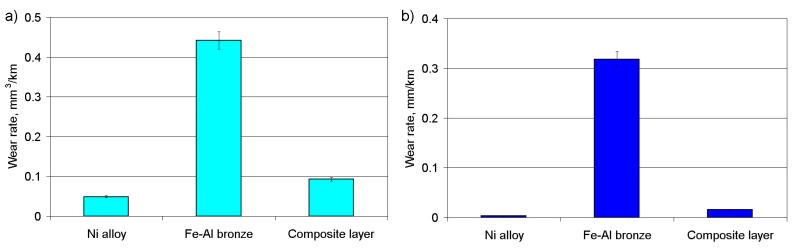
Comparing the wear rates under various friction conditions. (**a**) Dry friction, (**b**) boundary friction.

Comparing the wear rates under various friction conditions (Figure 11) and their costs according to the data of the metal exchange, it can be claimed that the composite coating under conditions of intensive wear will be economically profitable, in comparison with the use of the Ni alloy or bronze in its initial form. The first of the base materials (Ni alloy) has advantages in terms of its consumption over lengthy operating periods, but at the same time, it will be much more expensive in terms of its nominal cost and the surface finishing of the coating deposited. The second of the base materials (bronze), on the contrary, will be processed easily after deposition and has a lower cost, but its intensive wear will increase the number of regeneration cycles of the worn surfaces, the costs of assembling and the disassembling of friction units, etc.

### 3.5. Discussion

Recently, functionally graded materials (FGM) have become widespread. In these materials, compositions/functions and/or microstructures are gradually changing in one or more spatial directions. The changes lead to a gradual transformation of the material properties and functions. In particular, Kapil et al. [33] showed that bimetallic objects manufactured by a rapid tooling process reduced the manufacturing cycle time, improved the part integrity, and reduced the overall production cost through the use of cheaper materials. Shang et al. [34] analyzed FGMs based on TA15 and Inconel 718 alloys, particularly the peculiarities of crack formation, microstructure, and mechanical properties. Rajasekhar et al. [35] considered the interfacial properties of a double-layered Al–Cu functionally graded material including mechanical and microstructural properties and relative density. Meng et al. [36] studied FGM fabricated by the DED based on Inconel 625 and Ti6Al4V alloys, and focused i.a. on microstructure changes, material behavior during cracking, phase characteristics, and microhardness. Wang et al. [37] described FGM layers deposited on a Ti6Al4V alloy surface using laser cladding. Residual stress distribution in FGM layers was significantly affected by material properties.

Analyzing the studies described above, it should be emphasized that most frequently either authentic or quite similar materials have been applied as powder and substrate materials such as nickel superalloy and stainless steel. Obviously, in such cases, the problem of the quality connection of dissimilar materials is absent. In almost 25% of studies, the authors do not indicate the substrate material. In a very limited number of publications, a transition layer of a third material with chemical affinity to the base material has been used to ensure effective joining. This approach, however, is expensive and not always possible.

In this study, the possibilities of the effective formation of composite coating based on heterogeneous metal alloys deposited onto a steel substrate were considered. 12N-01 nickel alloy and Al–Fe bronze were used as initial coating materials; the 1045 structural steel was used as a substrate and direct metal deposition was used as the additive manufacturing method.

## 4. Conclusions

Based the analysis of the research results, the following results can be presented:

- The microstructure of the composite layer, deposited on the steel substrate, was very uniform. In particular, the bronze track, deposited between the tracks of Ni alloy, had a dendritic structure, which suggests a high level of crystallization. The Ni based alloy was characterized by its globular structure with the eutectic ingredient of the Ni alloy. When the depositing speed of the nickel based alloy increased, the thickness of the transition zone reduced and the dendrites passed to the quasi-eutectic state.
- Microhardness increased significantly at the interface between the substrate and the metals deposited. The transition zone extended into the substrate body and its thickness did not exceed 0.3 mm. At a distance of 0.1 mm from the substrate, the microhardness of the nickel alloy track nearly became constant throughout the whole coating thickness and was, on average, HV_100_ = 550. In the case of the bronze track, microhardness at the interface was close in value to the Ni alloy microhardness, but already at a distance of 0.3 mm from the interface, microhardness decreased to HV_100_ = 450 and remained at this level throughout the whole coating thickness.
- The microhardness of the coating layer depends on the depositing parameters. Increasing the cladding speed of Ni alloy reduced the microhardness, regardless of the cladding pitch. The microhardness of bronze in the composite coating did not depend on the cladding speed. The greatest microhardness, at all cladding speeds, was observed at the S_1_/S_2_ = 2.4/1.2 mm pitches. The changes indicated are due to changes in the volume of the molten pond and the hardening rate of the composite layer.
- During the running-in time of the coating layer under dry friction conditions, the CoF was less than 0.20 while in periods of stable wear, the CoF constantly increased to a level of 0.4–0.45, after which a period of accelerated wear was observed. There was an inversely proportional dependence between the values of the CoF and the wear rate under dry friction conditions. Under boundary friction conditions, where loading was increased by 120 to 240 N, the wear rate increased 2.2-fold, irrespective of the depositing conditions. At higher loads, the adhesion of coating material on the grain boundaries decreased and was destroyed.

Summarizing the above, it can be argued that if the components of the composite coating are chosen correctly, it is possible to obtain advantageous direct metal deposition conditions, thus ensuring a reliable and durable connection between themselves and the substrate. Based on this idea, it can be possible to produce functionally graded composite materials with high quality mechanical, structural and operation properties.

## Figures and Tables

**Table 1:** The chemical compositions of the 12N-01 nickel alloy and aluminum–iron bronze.

12N-01 Alloy Composition, %						Fe–Al Bronze Composition, %		
C	B	Si	Cr	Fe	Ni	Al	Fe	Cu
0.3–0.6	1.7–2.5	1.2–3.2	8–14	1.2–1.3	The rest	8.5–10.5	4	The rest

## Data Availability

Data sharing is not applicable to this article.
